# Expression of the CLCA4 Gene in Esophageal Carcinoma and Its Impact on the Biologic Function of Esophageal Carcinoma Cells

**DOI:** 10.1155/2021/1649344

**Published:** 2021-06-04

**Authors:** Xin Song, Shuai Zhang, Shouchuan Li, Ye Wang, Xinming Zhang, Feng Xue

**Affiliations:** Department of General Surgery, Qingdao Hospital of Traditional Chinese Medicine, Qingdao Hiser Hospital, Qingdao 266000, China

## Abstract

**Background:**

Esophageal carcinoma (ESCA) is one of the malignant tumors with a high mortality rate worldwide, which seriously affects people's health. Calcium-activated chloride channel 4 (CLCA4) was reported to be a tumor inhibitor in hepatocellular carcinoma. Nevertheless, the role of CLCA4 in ESCA is still unclear.

**Methods:**

RT-qPCR and western blot assay were used to test the expression pattern of CLCA4 in ESCA tissues and cells. CCK-8 assay was performed to detect the effect of CLCA4 overexpression on cell proliferation in ESCA cells. Transwell assay was used to measure the effect of CLCA4 upregulation on migration and invasion abilities of ESCA cells. Animal experiments were conducted to investigate the role of CLCA4 upregulation in tumor growth in vivo.

**Results:**

CLCA4 was significantly reduced in ESCA tissues and correlated with *T* stage, differentiation, and lymph node metastasis. CLCA4 overexpression was found to inhibit cell proliferation, migration, invasion, and EMT progression in ESCA cells. Moreover, CLCA4 overexpression suppressed tumor growth in vivo.

**Conclusion:**

CLCA4 was suggested to act as a tumor inhibitor in ESCA and might be a therapeutic target gene for the treatment of patients with ESCA.

## 1. Introduction

Esophageal carcinoma (ESCA) is one of the common digestive tract tumors, mainly including esophageal squamous cell carcinoma and esophageal adenocarcinoma [1]. The incidence of ESCA in China is high, accounting for 46.6% of the world's ESCA patients. According to research reports, the 5-year survival rate of patients with ESCA is only 15 ∼ 25% [2], which seriously affects people's health and life safety [3]. The occurrence and development of ESCA is a complex process including alcohol, smoking, dietary, multigene interaction [4, 5], and environmental factors [6]. At present, the treatment of ESCA mainly consists of surgery, radiotherapy, and chemotherapy, but the treatment effect is still not ideal, and the mortality rate is still high. Therefore, in-depth study on the pathogenesis of ESCA has become a hot topic, which provides a basis for early diagnosis, timely and effective treatment, and improving the survival rate of patients with ESCA.

The calcium-activated chloride channel (CLCA) gene family is located in the same region on chromosome p31-p22. The CLCA gene family has been reported to have a variety of functions, including cell adhesion, tumor inhibition, tumor promotion, and accessory molecules [7]. There are four kinds of CLCA regulatory proteins, namely, CLCA1, CLCA2, CLCA3, and CLCA4 [8]. CLCA1 is the first CLCA gene to be identified and cloned in human [9]. CLCA1 was reported to be closely related to the regulation of cell metastasis and immune invasion of colorectal cancer [10]. The CLCA2 gene can be used as a novel p53 inducible growth inhibitor, and downregulation can promote the proliferation of breast cancer cells [11]. CLCA3 may be involved in regulating the pathogenesis of allergic rhinitis [12]. CLCA4 has a similar cellular structure to CLCA2, and it has been found that CLCA4 plays a key role in EMT [13]. Yu et al. showed that CLCA4 was less expressed in breast cancer cells and CLCA4 downregulation promoted the proliferation of breast cancer cells and induced epithelial-mesenchymal transformation [13]. CLCA4 was found to repress cell migrated and invasive abilities by inhibiting the EMT pathway through the PI3K/AKT pathway in colorectal cancer [14]. Through a literature search, the expression and function of CLCA4 in esophageal cancer patients were not found.

In this study, through bioinformatics analysis, it was found that the expression of CLCA4 was low in esophageal cancer tissues. Moreover, the low expression of CLCA4 promoted the cell progression of esophageal cancer cells in vivo and in vitro. This study provides a theoretical basis for the development and prognosis of esophageal cancer.

## 2. Materials and Methods

### 2.1. Clinical Information

ESCA tissues and paracancerous tissues were collected from 84 patients at Qingdao Hospital of Traditional Chinese Medicine, Qingdao, Shandong, China. Among the 84 patients with ESCA (age: 36–72 years, mean: 54 years), 16 cases were female and 68 were male. All ESCA patients were diagnosed pathologically and had not received chemotherapy or radiotherapy before surgery. All patients have signed informed consent before surgery. This study was approved by the ethics committee of the Qingdao Hospital of Traditional Chines Medicine.

  Inclusion criteria: ① all patients were diagnosed with ESCA by pathological biopsy; ② all patients were initially treated and had no family genetic history
  Exclusion criteria: ① patients received radiotherapy, chemotherapy, or other targeted treatment before surgery; ② patients with other malignancies; and ③ patients with severe heart, liver, or renal insufficiency

### 2.2. Cell Culture and Cell Transfection

Esophageal epithelia cells HEEC and ESCA cell lines TE-1, Eca-109, TE-10, EC9706, and KYSE-150 were purchased from Shanghai JingKang Bioengineering CO., LTD. (Shanghai, China). All cells were cultured in the Roswell Park Memorial Institute 1640 (RPMI-1640) medium with 10% FBS at 37°C in a 5% CO_2_ incubator.

Eca-109 cells and KYSE-150 cells were placed in a 6-well plate and infected with pcDNA3.1 vector or pcDNA3.1-CLCA4 vector. Cell transfection was performed by Lipofectamine 2000 according to the manufacturer's instructions. After infection for 72 h, cells were collected to detect the transfection efficiency by using RT-qPCR or for other experiments.

### 2.3. RT-qPCR Analysis

RT-qPCR analysis was used to detect the expression level of CLCA4 mRNA in ESCA tissues and cells. Total RNA was extracted by adding Trizol lysate. RNA concentration and purity were detected by using an ultraviolet spectrophotometer, and the RNA was stored in a −80°C refrigerator. The reaction system was configured according to the instructions of the reverse transfection kit, and the cDNA was obtained by PCR amplification. Subsequently, Ct values of each group were detected by RT-qPCR. GAPDH was used as the internal reference gene of CLCA4. The 2^−ΔΔCt^ method was carried out to calculate the relative mRNA expression of CLCA4. All primers were designed and synthesized by Shanghai Sangon Biological Engineering Co., Ltd. (Shanghai, China). The primer sequences used in this study were GAPDH, forward: 5′-TCCTCTGACTTCAACAGCGACAC-3′; reverse: 5′-TCTCTCTTCCTCTTGTGCTCTTGC-3′ and CLCA4 forward: 5′-TTTGGGGCTCTTACATCAGG-3′; reverse: 5′-GTGTCGTTCATCCAGGCATT-3′.

### 2.4. Western Blot Assay

Cells (1 × 10^7^ cells/mL) were lysed with 100–150 *μ*L lysate solution on ice for 1 h. Then, proteins were collected by centrifugation at low temperature and high speed. The protein concentration was determined by BCA protein quantitative assay. 30 *μ*g protein was mixed with SDS buffer and boiled at 100°C for 5 min for denaturation. After 10% Sodium Dodecyl Sulfate Polyacrylamide Gel Electrophoresis (SDS-PAGE), proteins were transferred to the PVDF membrane. After blocked with 5% skim milk for 2 h, the membrane was incubated with the primary antibody at 37°C for 2 h. After washed with TBST solution 3 times, the membrane was incubated with the corresponding secondary antibody for 1 h. After washing with TBST solution 3 times, the protein expression of CLCA4 was detected by using the luminescence image system.

### 2.5. Cell Counting Kit-8 (CCK-8) Assay

Cell proliferation was measured by CCK-8 assay. 100 *μ*L cells (1 × 10^5^ cells/mL) were inoculated on a 96-well plate. 6 duplicate wells were set in the same detection time group. The plate was placed into an incubator (37°C and 5% CO_2_). Then, 10 *μ*L CCK-8 reagent was added to each group at 0, 24, 48, 72 h, respectively. The cells were cultured at 37°C for 1–4 h, until the color of the medium changed. The optical density of each well was measured by using a microplate analyzer at 450 nm.

### 2.6. Transwell Assay

ESCA cell motility was detected by Transwell assay. The frozen Matrigel was liquefied and diluted (1 mg/mL) and then added into the Transwell chamber. After incubation at 37°C for 1 h, the chamber was washed with the medium for 3 times. 100 *μ*L cell suspensions (2.5 × 10^4^ cells/mL) was added into the upper chamber previously uncoated with Matrigel (for the migration test) or coated with Matrigel (for the invasion test), respectively. The lower chamber was added with normal Dulbecco's Modified Eagle's Medium (DMEM) and cultured for 24 h (5% CO_2_ and 37°C). The cells were absorbed, then fixed with paraformaldehyde, and stained with crystal violet. After rinsed with Phosphate Buffer Saline (PBS), the number of migrated or invaded cells was counted under a light microscope. Five fields were randomly selected to calculate the average value of each well.

### 2.7. Animal Experiments

The effect of CLCA4 on tumor growth in vivo was detected by the tumor xenograft model. Male BALB/C nude mice were obtained from Shanghai Slack Laboratory Anima Co., Ltd. (Shanghai, China). Eca-109 cells transfected with CLCA4 vector or vector were isolated by 0.25% trypsin and resuspended in phosphate buffered saline. Then, the cell suspension was injected into the flank of mice subcutaneously. The transplanted tumors were weighed and measured weekly. All mice were euthanized 5 weeks after inoculation. Animal experiments were carried out in accordance with the Animal Protection Law of the People's Republic of China-2009.

### 2.8. Statistical Analysis

Data were analyzed by SPSS20.0 and GraphPad Prism 7.0 software. All data graphs were drawn by GraphPad Prism 7.0 software. The relationship between CLCA4 expression and clinicopathological data in patients with ESCA was determined by the rank-sum test. Differences between the two groups were detected by Student's *t*-test. *p* < 0.05 was considered statistically significant.

## 3. Results

### 3.1. CLCA4 Expression is Obviously Declined in ESCA

**Figure 1:**
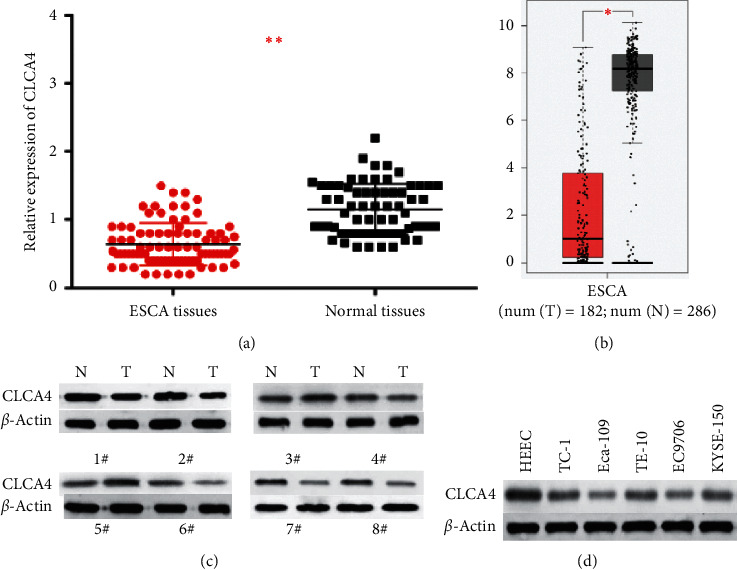
CLCA4 expression is obviously declined in ESCA. (a) The expression of CLCA4 in ESCA tissues was detected by RT-qPCR. (b) The GEPIA database displayed that the expression of CLCA4 was low in ESCA tissues. (c) The protein expression of CLCA4 in 8 ESCA tissues was detected by western blot. (d) The protein expression of CLCA4 in ESCA cells. ^*∗∗*^*p* < 0.01.

In order to identify the role of CLCA4 in ESCA, the expression level of CLCA4 was first tested in ESCA tissues and cells by RT-qPCR and western blot assay. RT-qPCR results indicated that CLCA4 expression was dramatically lower in ESCA tissues than in nontumor tissues (Figure 1(a)). Likewise, bioinformatics analysis displayed that CLCA4 was downregulated in ESCA tissues compared to the normal tissues (Figure 1(b)). Western blot assay showed that CLCA4 protein expression was downregulated in 69.05% (58/84) of ESCA samples (Figure 1(c)). As expected, we found that CLCA4 expression was lower in TE-1, Eca-109, TE-10, EC9706, and KYSE-150 cells than in esophageal epithelial cells HEEC (Figure 1(d)).

To evaluate the effect of CLCA4 in ESCA patients, the correlation between CLCA4 expression and clinicopathological factors in ESCA patients was analyzed. As shown in Table 1, the expression of CLCA4 was closely correlated with *T* stage (*p*=0.024), differentiation (*p*=0.039), and lymph node metastasis (*p*=0.048). Inversely, CLCA4 expression was not significantly associated with tumor size (*p*=0.575), age (*p*=0.513), and gender (*p*=0.671). Furthermore, univariate and multivariate logistic analyses were performed. The results displayed that CLCA4 expression was significantly related to differentiation (Table 2). Hence, our data indicated that CLCA4 was downregulated in ESCA and might be a diagnostic marker of ESCA.

### 3.2. CLCA4 Overexpression Inhibits Cell Viability and EMT Progression in ESCA Cells

**Figure 2:**
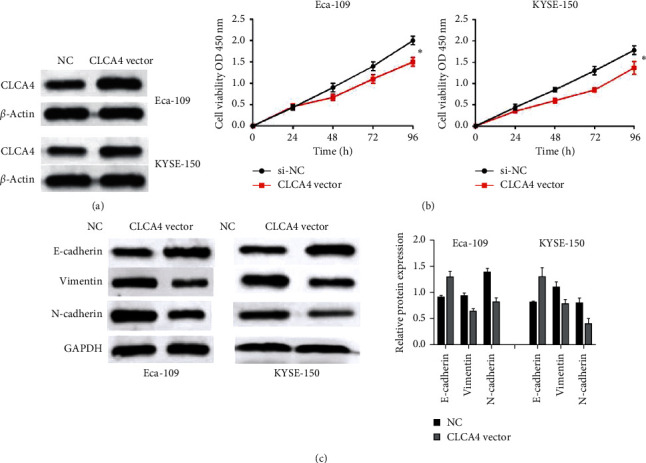
CLCA4 overexpression inhibits cell viability and EMT progression in ESCA cells. (a) The expression of CLCA4 in KYSE-150 and Eca-109 cells with CLCA4 vector. (b) Cell proliferation in KYSE-150 and Eca-109 cells with CLCA4 vector was detected by CCK-8 assay. (c) EMT progression in KYSE-150 and Eca-109 cells with CLCA4 vector was detected by western blot. ^*∗*^*p* < 0.05; ^*∗∗*^*p* < 0.01.

Next, the biological function of CLCA4 on the progression of ESCA was detected. We transfected CLCA4 vector into KYSE-150 and Eca-109 cells. The transfection efficiency was detected by RT-qPCR assay. We noticed that the expression of CLCA4 was obviously elevated in KYSE-150 and Eca-109 cells after transfection with CLCA4 vector (Figure 2(a)). Then, cell proliferation of Eca-109 and KYSE-150 cells was explored by MTT assay. Overall, ESCA cell proliferation was significantly inhibited by overexpression of CLCA4 compared with the control group (Figure 2(b)). To investigate the effect of CLCA4 on EMT progression, western blot assay was carried out to detect the relative expressions of EMT-related proteins (E-cadherin, E-cadherin, and vimentin). Upregulation of E-cadherin and downregulation of vimentin and N-cadherin were detected in Eca-109 and KYSE-150 cells transfected with CLCA4 overexpression (Figure 2(c)). These findings indicated that CLCA4 might restrain cell viability and EMT progression in ESCA cells.

### 3.3. CLCA4 Overexpression Inhibits Cell Migration and Invasion Capabilities in ESCA Cells

**Figure 3:**
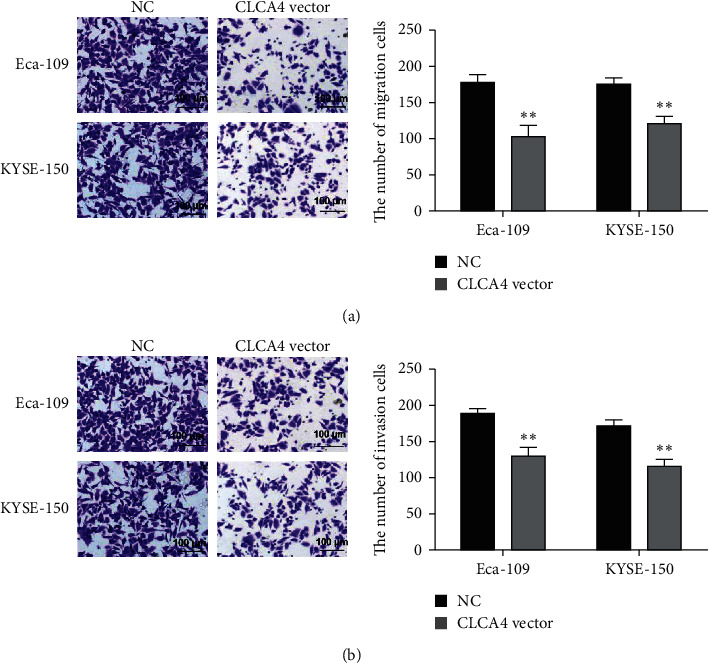
CLCA4 overexpression inhibits cell migration and invasion capabilities in ESCA cells. (a) Cell migration was inhibited in KYSE-150 and Eca-109 cells with CLCA4 vector (scale bar = 100 *μ*m). (b) Cell invasion was inhibited in KYSE-150 and Eca-109 cells with CLCA4 vector (scale bar = 100 *μ*m). ^*∗∗*^*p* < 0.01.

Furthermore, cell migration and invasion in ESCA cells were measured by Transwell assay. In the migration experiment, after incubation in a serum-free medium for 24 h, we found that the number of migrated cells was remarkably reduced in KYSE-150and Eca-109 cells with CLCA4 vector (Figure 3(a)). Similarly, CLCA4 overexpression obviously decreased the cell invasion ability of KYSE-150 and Eca-109 cells through the basement membrane (Figure 3(b)). All data suggested that overexpression of CLCA4 might decrease the abilities of cell migration and invasion in ESCA cells.

### 3.4. CLCA4 Overexpression Impairs Tumorigenicity of ESCA Cells

**Figure 4:**
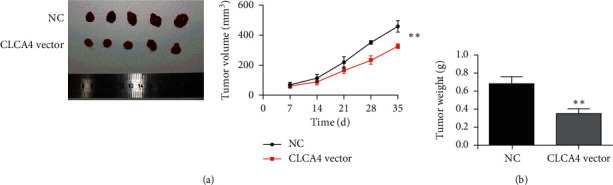
CLCA4 overexpression impairs tumorigenicity of ESCA cells. (a) CLCA4 overexpression suppressed tumor growth in vivo. (b) The weight of tumors with CLCA4 overexpression was dramatically smaller than in the control group. ^*∗∗*^*p* < 0.01.

To explore the function of CLCA4 on the tumorigenicity of ESCA cells, the tumor xenograft experiment was performed. Eca-109 cells transfected with CLCA4 vector or vector were injected into the flank of nude mice. Every 7 days, the growth conditions of the tumors were recorded. We noticed that the tumors with CLCA4 overexpression (323.33 ± 25.17 mm^3^) were dramatically smaller than the control group (460.72 ± 36.06 mm^3^) after 5 weeks (Figure 4(a)). After 5 weeks, all mice were euthanized, and the weight of CLCA4 vector tumors (0.68 ± 0.076 g) was dramatically smaller than in the control group (0.35 ± 0.095 g) (Figure 4(b)). Therefore, our results confirmed that the upregulation of CLCA4 inhibited tumorigenicity in ESCA cells.

## 4. Discussion

ESCA is one of the common malignant tumors that endanger human health. The incidence of ESCA is the tenth most common cancer worldwide, and the mortality rate of ESCA is the sixth in the world [15]. China is a region with a high incidence of ESCA, accounting for 53.7% of the global incidence of ESCA [16]. Although the incidence of ESCA has declined in recent years, the death rate remains high. In consequence, it is of great significance to further study the mechanism for early diagnosis and survival rate improvement of ESCA. Studying the pathogenesis of ESCA at the genetic level can provide some theoretical basis for the diagnosis and treatment of ESCA.

Researchers have found that multiple genes are closely related to the occurrence and development of human cancers [17]. Wang et al. reported that SPINK5 acted as a tumor suppressor by suppressing the Wnt/beta-catenin pathway in ESCA [18]. Lan et al. proved that TRPM8 facilitated cell growth and immune evasion in ESCA cells [19]. RSRC2 was proved to be a tumor inhibitor and a prognostic target gene inESCA [20]. In this article, we found that CLCA4 was obviously downregulated in ESCA tissues and cells. Simultaneously, CLCA4 expression was negatively correlated with T stage, differentiation, and lymph node metastasis. Similar to our results, Hou et al. confirmed that CLCA4 was at a low level in bladder cancer [21]. Moreover, CLCA4 was reduced in colorectal cancer, and CLCA4 expression was associated with the overall survival rate of patients with breast cancer, stomach cancer, colorectal cancer, and head and neck cancer [22].

CLCA4 was reported to inhibited cell multiplication and metastasis by inhibiting the PI3K/AKT signaling pathway in bladder cancer [21]. CLCA4 was found to act as a tumor suppressor gene in some cancers, but its role in ESCA is still unclear. To investigate the role of CLCA4 in ESCA, the functional experiments were performed by Eca-109 and KYSE-150 cells. CCK-8 assay and Transwell assay were used to explore the effect of CLCA4 on cell proliferation, migration, and invasion abilities. We found that CLCA4 overexpression obviously suppressed cell proliferation, cell migration, cell invasion, and EMT progression in ESCA cells. Therefore, our findings confirmed that CLCA4 might block tumor growth and motility in ESCA. In line with our findings, CLCA4 was found to block cell migration and cell invasion by inhibiting the EMT pathway through the PI3K/ATK pathway in hepatocellular carcinoma [23]. CLCA4 overexpression was found to significantly decline the cell proliferation and metastasis ability in head and neck squamous cell carcinoma cells [24]. Furthermore, CLCA4 might be a target gene for primary colorectal cancer [25]. In addition, the tumor xenograft experiment was performed to detect the function of CLCA4 on the tumorigenicity in vivo. We found that both the weight and volume of tumors were significantly smaller than in the control group.

## 5. Conclusions

As far as we know, this is the first study to investigate the role of CLCA4 in ESCA. Our study verified that CLCA4 expression was obviously declined in ESCA tissues. Besides, the inhibitory effect of CLCA4 overexpression on cell progression in vivo and in vitro had also confirmed it. However, more studies are needed to confirm the specific mechanism of CLCA4 in ESCA. Collectively, our study confirmed that CLCA4 played a role as an antioncogene in ESCA and could provide a basis for a potential treatment approach for ESCA patients.

## Figures and Tables

**Table 1:** Association between CLCA4 expression and clinicopathological characteristics of patients with ESCA.

	Number of cases	CLCA4 expression		*p* value
		Low (*n* = 46)	High (*n* = 38)	
Age (years)				0.513
<60	30	15	15	
≥60	54	31	23	

Gender				0.671
Female	16	8	8	
Male	68	38	30	

Tumor size				0.757
<5 cm	56	30	26	
≥5 cm	28	16	12	

T stage				0.024^*∗*^
I-II	55	35	20	
III-IV	29	11	18	

Differentiation				0.039^*∗*^
Moderate/high	34	14	20	
Low	50	32	18	

Lymph node metastasis				0.048^*∗*^
Present	27	19	8	
Absent	57	27	30	

^*∗*^ *p* < 0.05.

**Table 2:** Univariate and multivariate Cox regression models for estimating the overall survival.

Variable	Univariate analysis		Multivariate analysis	
	HR (95% CI)	*p* value	HR (95% CI)	*p* value
Age (<60 vs. ≥60)	0.514 (0.347–1.647)	0.628	2.314 (1.345–3.547)	0.884
Tumor size (<5 vs. ≥5)	0.422 (0.214–0.847)	0.254	0.363 (0.094–0.921)	0.075
Gender (male vs. female)	0.647 (0.396–1.474)	0.341	0.421 (0.143–1.258)	0.069
T stage (I-II vs. III-IV)	1.671 (1.024–2.364)	0.044	0.694 (0.232–2.547)	0.055
Differentiation (moderate/high vs. low)	0.347 (0.127–0.843)	0.128	0.087 (0.029–0.647)	0.023^*∗*^
Lymph node metastasis (present vs. absent)	1.694 (1.235–2.684)	0.034^*∗*^	0.987 (0.348–1.874)	0.058
CLCA4 (low vs. high)	1.549 (1.146–2.647)	0.024^*∗*^	1.124 (0.654–2.367)	0.018^*∗*^

HR: hazard ratio; CI: confidence interval; ^*∗*^*p* < 0.05, ^*∗∗*^*p* < 0.01.

## Data Availability

The data used to support the findings of this study are available from the corresponding author upon request.
